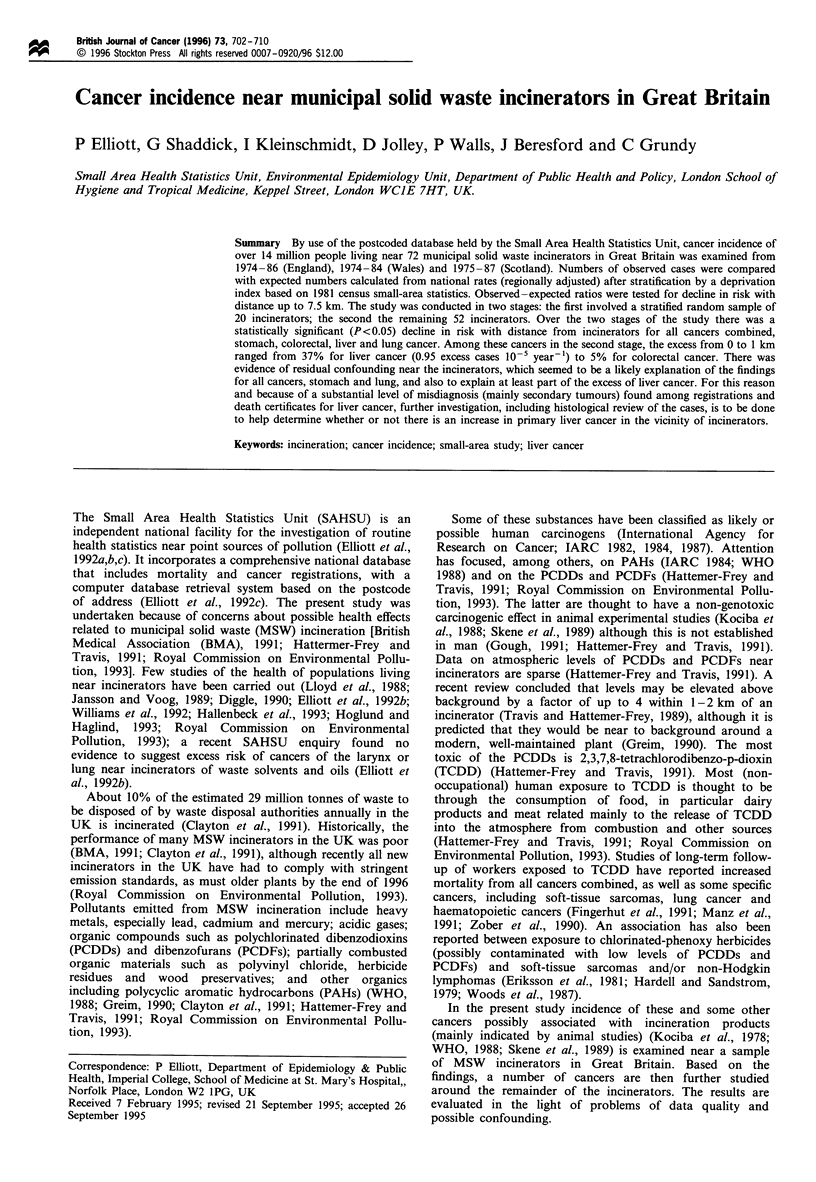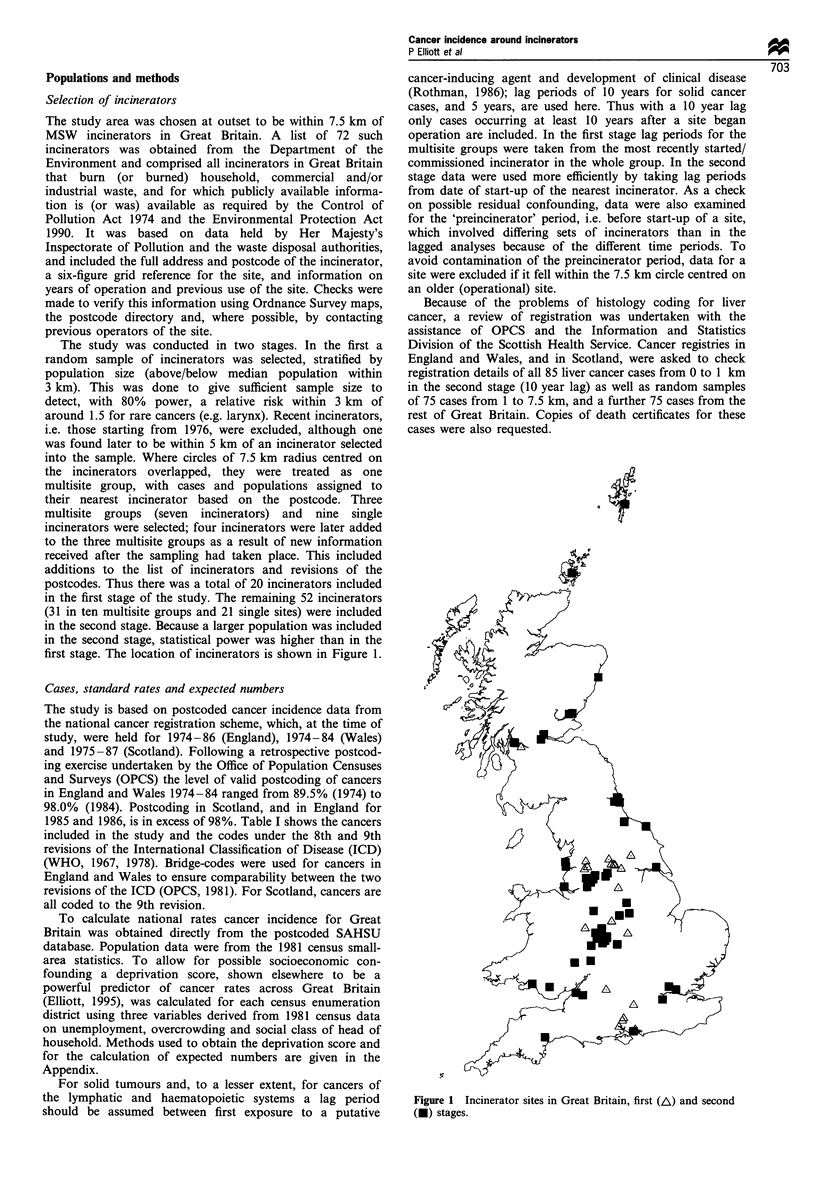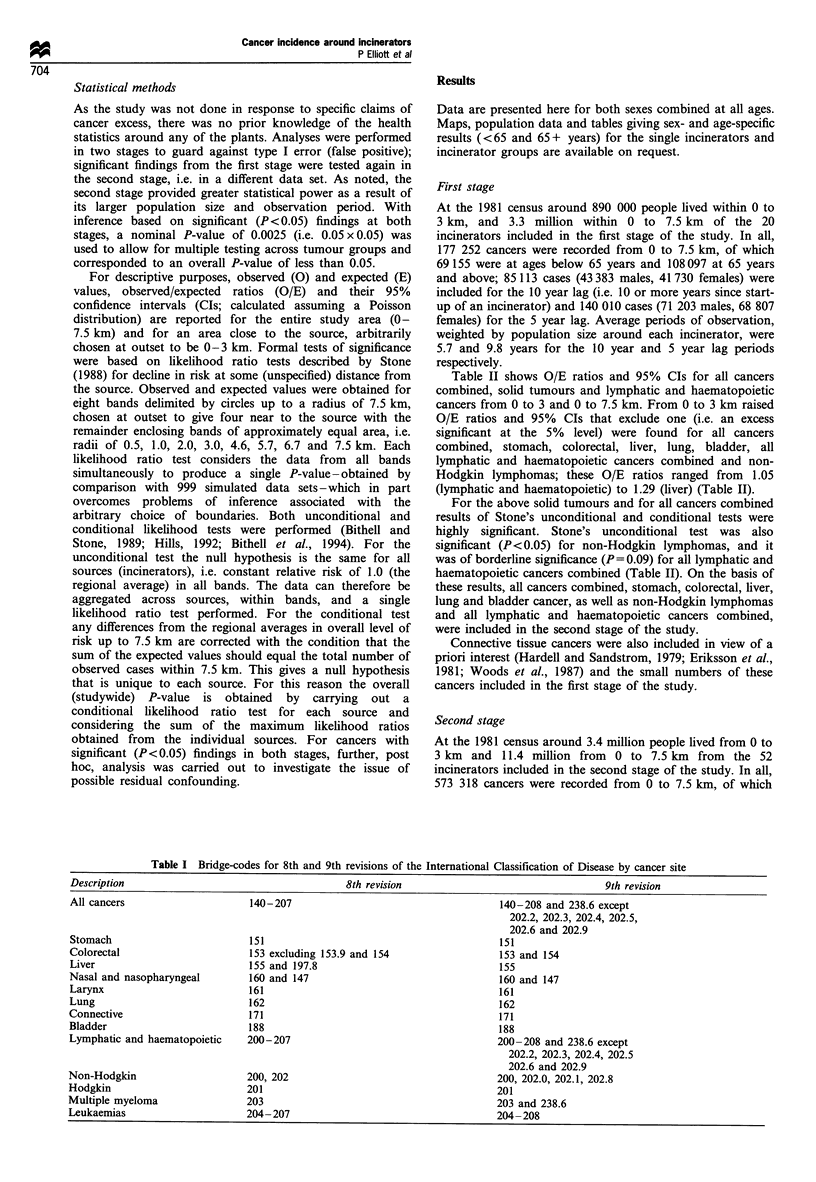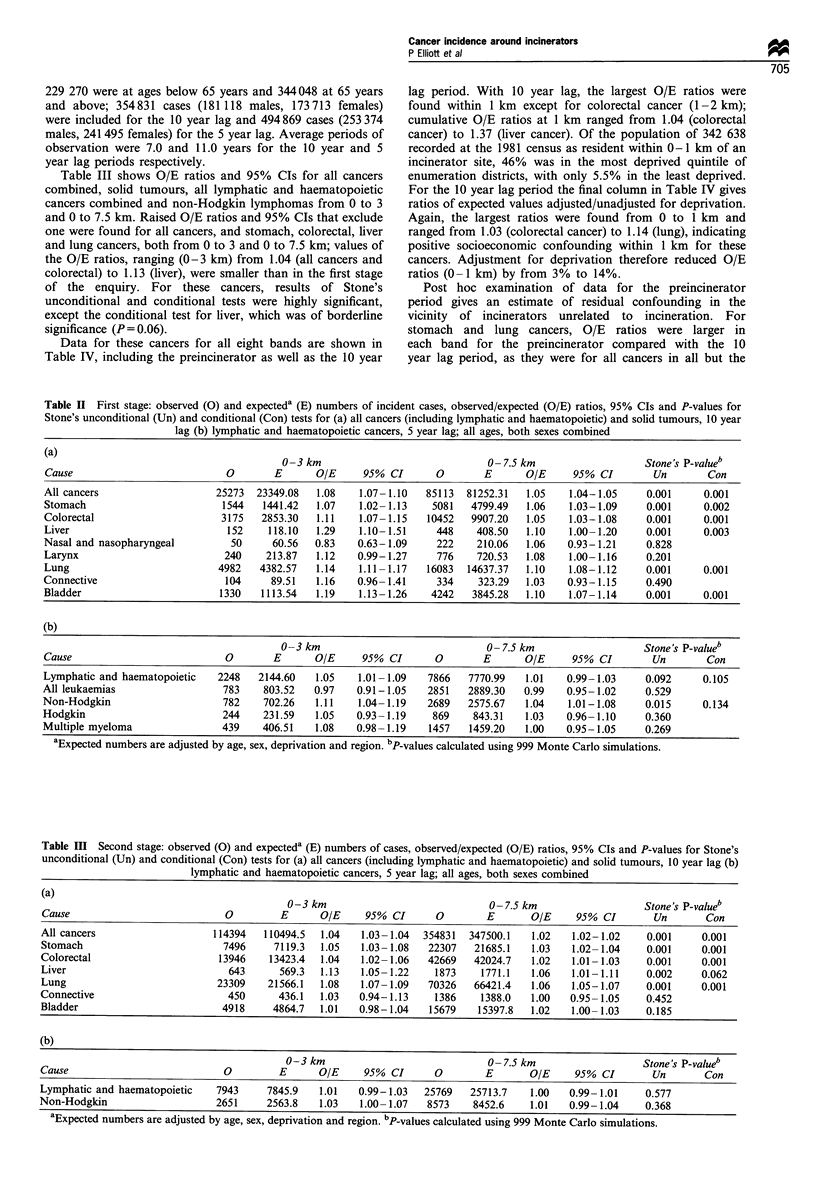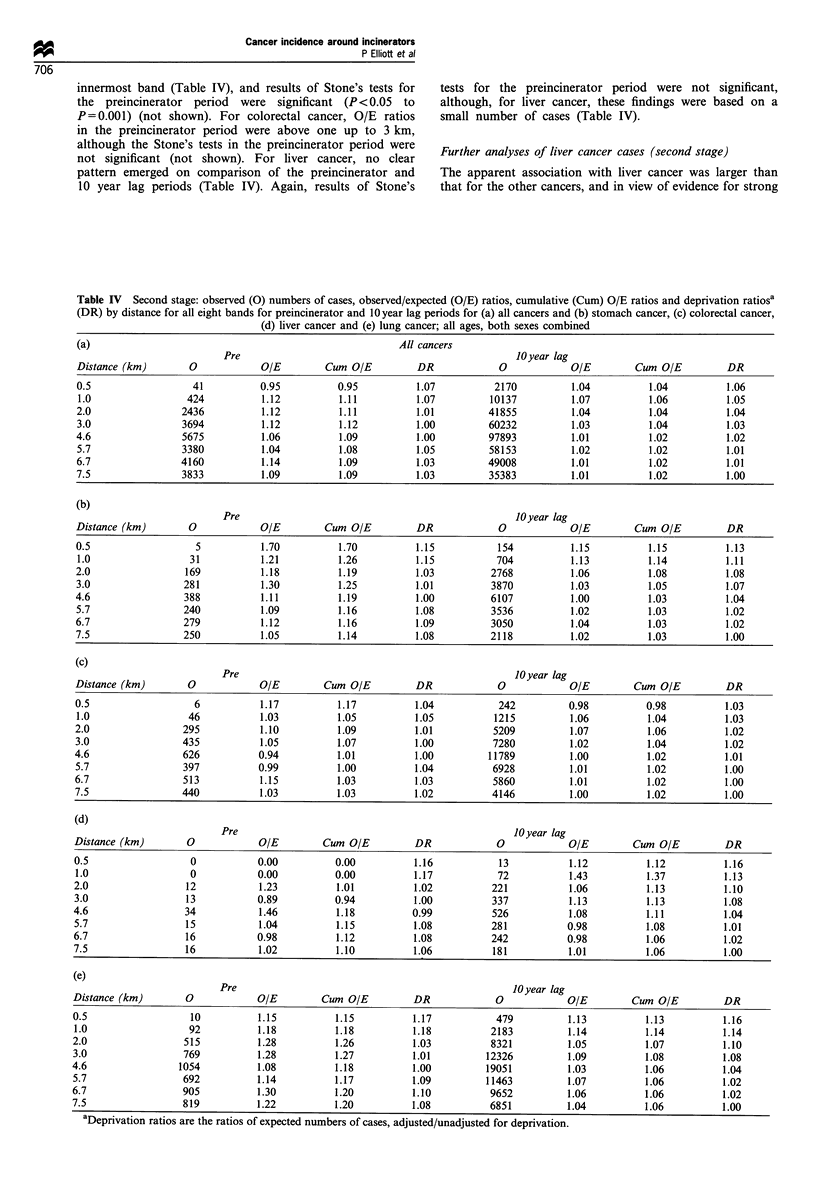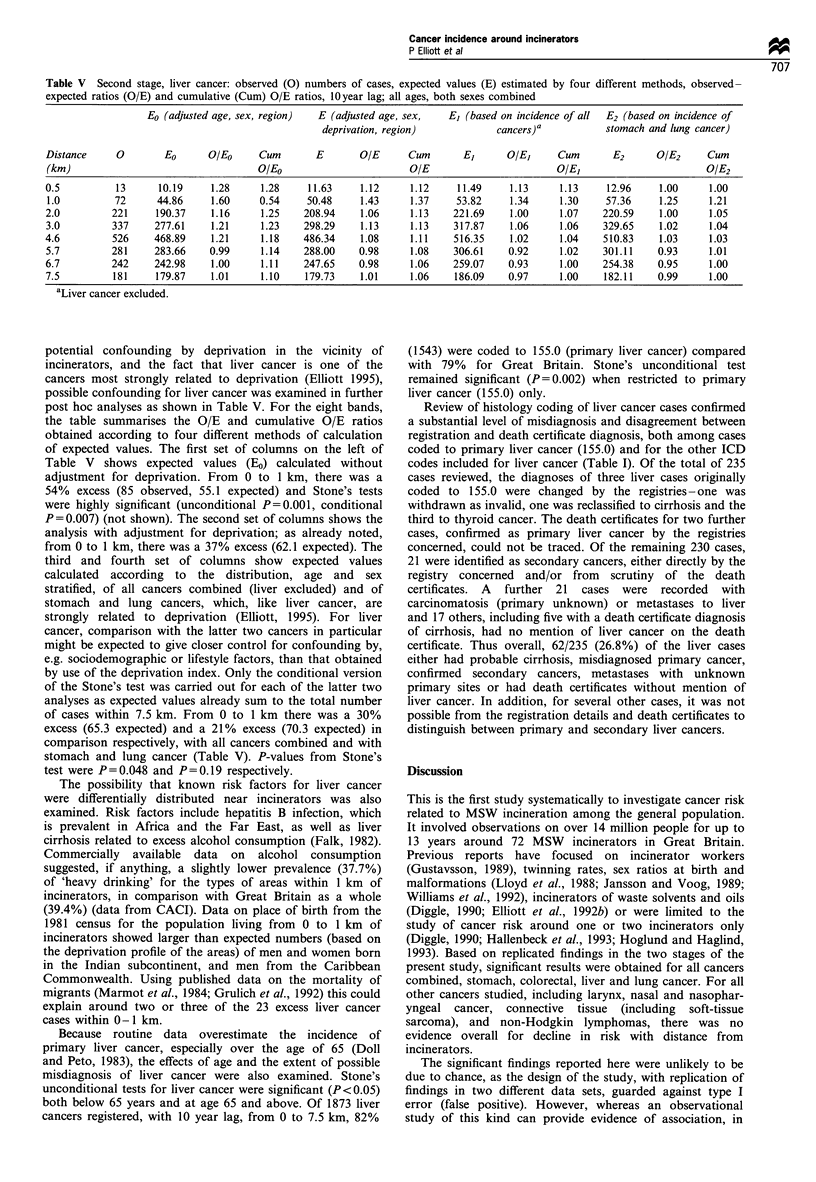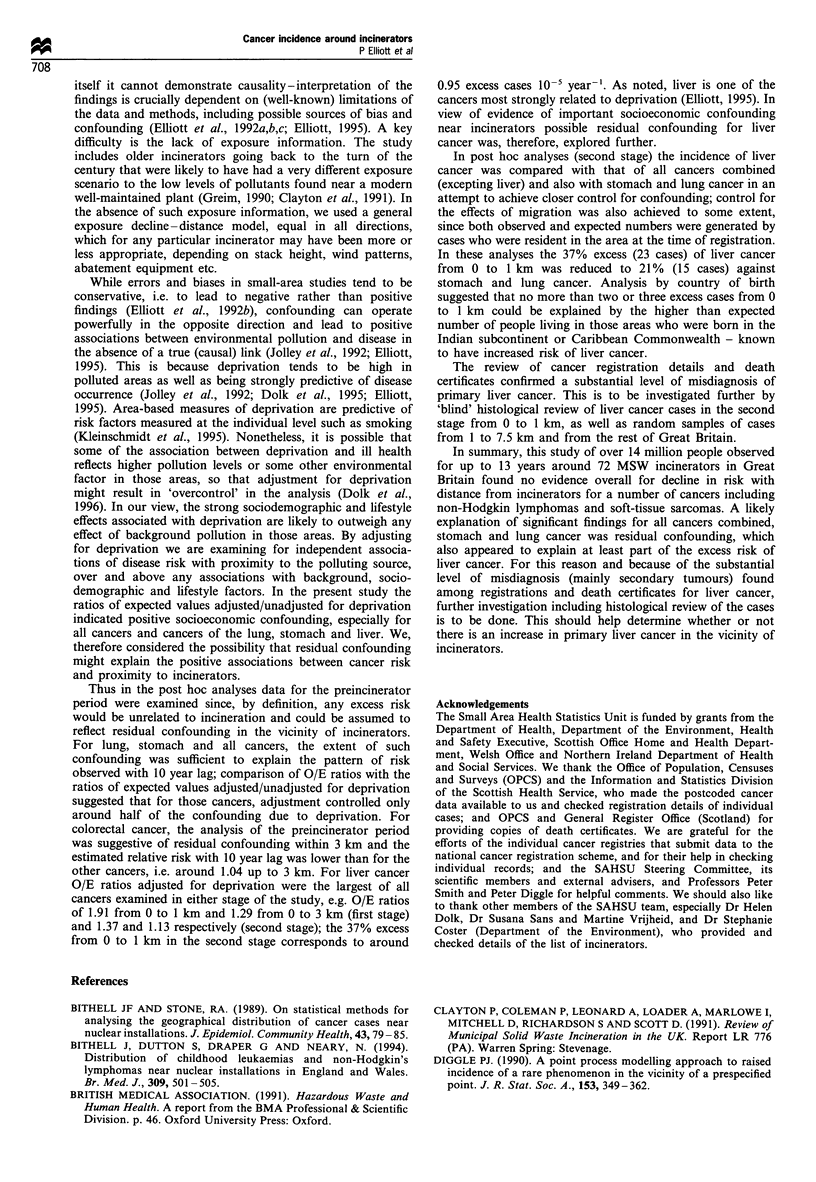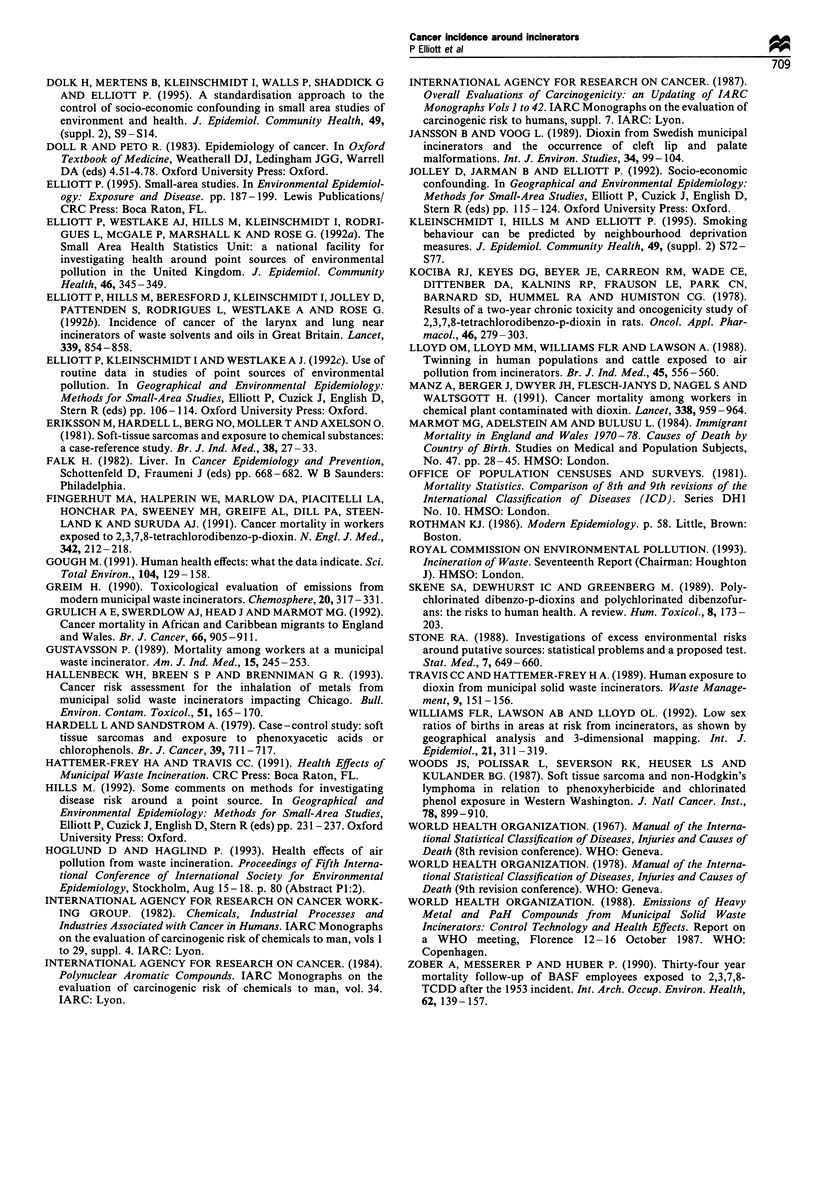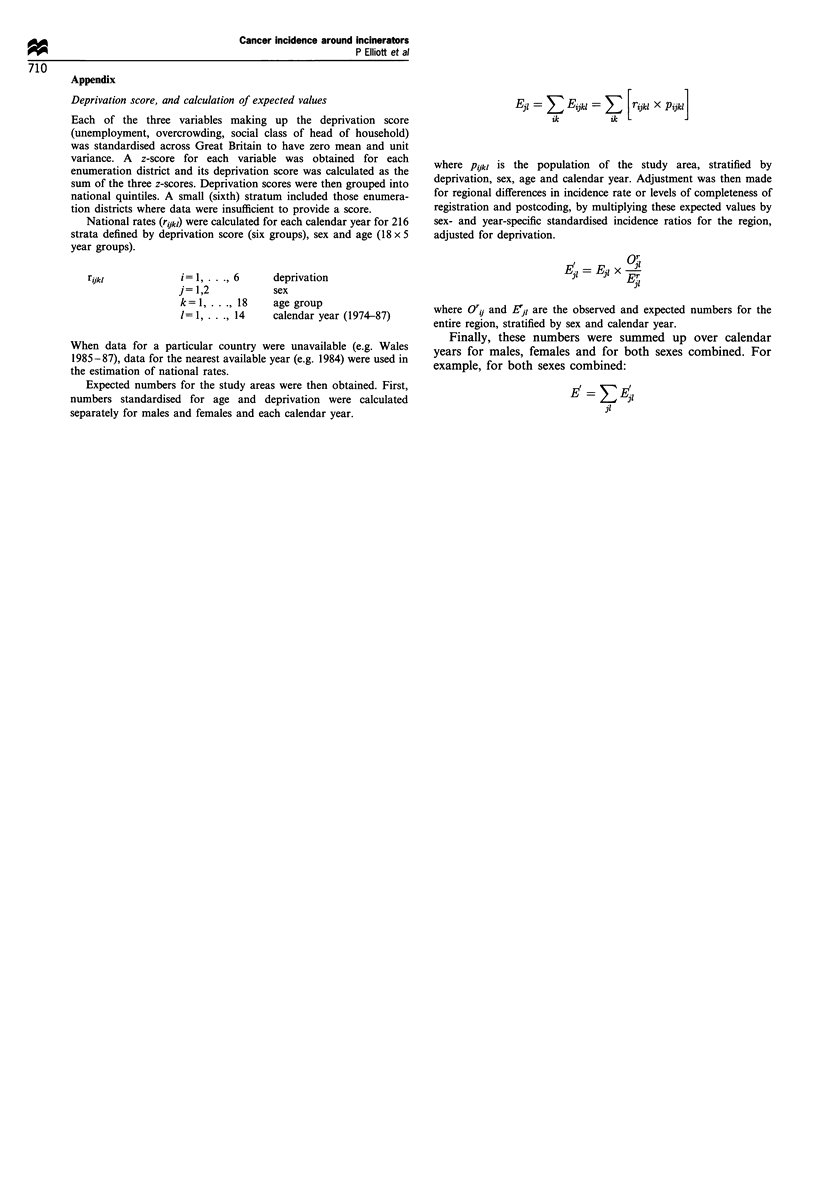# Cancer incidence near municipal solid waste incinerators in Great Britain.

**DOI:** 10.1038/bjc.1996.122

**Published:** 1996-03

**Authors:** P. Elliott, G. Shaddick, I. Kleinschmidt, D. Jolley, P. Walls, J. Beresford, C. Grundy

**Affiliations:** Small Area Health Statistics Unit, Department of Public Health and Policy, London School of Hygiene and Tropical Medicine, UK.

## Abstract

By use of the postcoded database held by the Small Area Health Statistic Unit, cancer incidence of over 14 million people living near 72 municipal solid waste incinerators in Great Britain was examined from 1974-86 (England), 1974-84 (Wales) and 1975-87 (Scotland). Numbers of observed cases were compared with expected numbers calculated from national rates (regionally adjusted) after stratification by a deprivation index based on 1981 census small area statistics. Observed-expected ratios were tested for decline in risk with distance up to 7.5 km. The study was conducted in two stages: the first involved a stratified random sample of 20 incinerators; the second the remaining 52 incinerators. Over the two stages of the study was a statistically significant (P<0.05) decline in risk with distance from incinerators for all cancers combined, stomach, colorectal, liver and lung cancer. Among these cancers in the second stage, the excess from 0 to 1 km ranged from 37% for liver cancer (0.95) excess cases 10(-5) per year to 5% for colorectal cancer. There was evidence of residual confounding near the incinerators, which seems to be a likely explanation of the finding for all cancers, stomach and lung, and also to explain at least part of the excess of liver cancer. For this reason and because of a substantial level of misdiagnosis (mainly secondary tumours) found among registrations and death certificates for liver cancer, further investigation, including histological review of the cases, is to be done to help determine whether or not there is an increase in primary liver cancer in the vicinity of incinerators.


British Journal of Cancer (1996) 73, 702–710
© 1996 Stockton Press All rights reserved 0007–0920/96 $12.00

### Cancer incidence near municipal solid waste incinerators in Great Britain

P Elliott, G Shaddick, I Kleinschmidt, D Jolley, P Walls, J Beresford and C Grundy

*Small Area Health Statistics Unit, Environmental Epidemiology Unit, Department of Public Health and Policy, London School of Hygiene and Tropical Medicine, Keppel Street, London WC1E 7HT, UK.*

**Summary** By use of the postcoded database held by the Small Area Health Statistics Unit, cancer incidence of over 14 million people living near 72 municipal solid waste incinerators in Great Britain was examined from 1974–86 (England), 1974–84 (Wales) and 1975–87 (Scotland). Numbers of observed cases were compared with expected numbers calculated from national rates (regionally adjusted) after stratification by a deprivation index based on 1981 census small-area statistics. Observed–expected ratios were tested for decline in risk with distance up to 7.5 km. The study was conducted in two stages: the first involved a stratified random sample of 20 incinerators; the second the remaining 52 incinerators. Over the two stages of the study there was a statistically significant ($P<0.05$) decline in risk with distance from incinerators for all cancers combined, stomach, colorectal, liver and lung cancer. Among these cancers in the second stage, the excess from 0 to 1 km ranged from 37% for liver cancer (0.95 excess cases $10^{-5}$ year$^{-1}$) to 5% for colorectal cancer. There was evidence of residual confounding near the incinerators, which seemed to be a likely explanation of the findings for all cancers, stomach and lung, and also to explain at least part of the excess of liver cancer. For this reason and because of a substantial level of misdiagnosis (mainly secondary tumours) found among registrations and death certificates for liver cancer, further investigation, including histological review of the cases, is to be done to help determine whether or not there is an increase in primary liver cancer in the vicinity of incinerators.

**Keywords:** incineration; cancer incidence; small-area study; liver cancer

The Small Area Health Statistics Unit (SAHSU) is an independent national facility for the investigation of routine health statistics near point sources of pollution (Elliott *et al.*, 1992*a*,*b*,*c*). It incorporates a comprehensive national database that includes mortality and cancer registrations, with a computer database retrieval system based on the postcode of address (Elliott *et al.*, 1992*c*). The present study was undertaken because of concerns about possible health effects related to municipal solid waste (MSW) incineration [British Medical Association (BMA), 1991; Hattermer-Frey and Travis, 1991; Royal Commission on Environmental Pollution, 1993]. Few studies of the health of populations living near incinerators have been carried out (Lloyd *et al.*, 1988; Jansson and Voog, 1989; Diggle, 1990; Elliott *et al.*, 1992*b*; Williams *et al.*, 1992; Hallenbeck *et al.*, 1993; Hoglund and Haglind, 1993; Royal Commission on Environmental Pollution, 1993); a recent SAHSU enquiry found no evidence to suggest excess risk of cancers of the larynx or lung near incinerators of waste solvents and oils (Elliott *et al.*, 1992*b*).

About 10% of the estimated 29 million tonnes of waste to be disposed of by waste disposal authorities annually in the UK is incinerated (Clayton *et al.*, 1991). Historically, the performance of many MSW incinerators in the UK was poor (BMA, 1991; Clayton *et al.*, 1991), although recently all new incinerators in the UK have had to comply with stringent emission standards, as must older plants by the end of 1996 (Royal Commission on Environmental Pollution, 1993). Pollutants emitted from MSW incineration include heavy metals, especially lead, cadmium and mercury; acidic gases; organic compounds such as polychlorinated dibenzodioxins (PCDDs) and dibenzofurans (PCDFs); partially combusted organic materials such as polyvinyl chloride, herbicide residues and wood preservatives; and other organics including polycyclic aromatic hydrocarbons (PAHs) (WHO, 1988; Greim, 1990; Clayton *et al.*, 1991; Hattemer-Frey and Travis, 1991; Royal Commission on Environmental Pollution, 1993).

Correspondence: P Elliott, Department of Epidemiology & Public Health, Imperial College, School of Medicine at St. Mary's Hospital,, Norfolk Place, London W2 1PG, UK
Received 7 February 1995; revised 21 September 1995; accepted 26 September 1995

Some of these substances have been classified as likely or possible human carcinogens (International Agency for Research on Cancer; IARC 1982, 1984, 1987). Attention has focused, among others, on PAHs (IARC 1984; WHO 1988) and on the PCDDs and PCDFs (Hattemer-Frey and Travis, 1991; Royal Commission on Environmental Pollution, 1993). The latter are thought to have a non-genotoxic carcinogenic effect in animal experimental studies (Kociba *et al.*, 1988; Skene *et al.*, 1989) although this is not established in man (Gough, 1991; Hattemer-Frey and Travis, 1991). Data on atmospheric levels of PCDDs and PCDFs near incinerators are sparse (Hattemer-Frey and Travis, 1991). A recent review concluded that levels may be elevated above background by a factor of up to 4 within 1–2 km of an incinerator (Travis and Hattemer-Frey, 1989), although it is predicted that they would be near to background around a modern, well-maintained plant (Greim, 1990). The most toxic of the PCDDs is 2,3,7,8-tetrachlorodibenzo-p-dioxin (TCDD) (Hattemer-Frey and Travis, 1991). Most (non-occupational) human exposure to TCDD is thought to be through the consumption of food, in particular dairy products and meat related mainly to the release of TCDD into the atmosphere from combustion and other sources (Hattemer-Frey and Travis, 1991; Royal Commission on Environmental Pollution, 1993). Studies of long-term follow-up of workers exposed to TCDD have reported increased mortality from all cancers combined, as well as some specific cancers, including soft-tissue sarcomas, lung cancer and haematopoietic cancers (Fingerhut *et al.*, 1991; Manz *et al.*, 1991; Zober *et al.*, 1990). An association has also been reported between exposure to chlorinated-phenoxy herbicides (possibly contaminated with low levels of PCDDs and PCDFs) and soft-tissue sarcomas and/or non-Hodgkin lymphomas (Eriksson *et al.*, 1981; Hardell and Sandstrom, 1979; Woods *et al.*, 1987).

In the present study incidence of these and some other cancers possibly associated with incineration products (mainly indicated by animal studies) (Kociba *et al.*, 1978; WHO, 1988; Skene *et al.*, 1989) is examined near a sample of MSW incinerators in Great Britain. Based on the findings, a number of cancers are then further studied around the remainder of the incinerators. The results are evaluated in the light of problems of data quality and possible confounding.

### Populations and methods

*Selection of incinerators*

The study area was chosen at outset to be within 7.5 km of MSW incinerators in Great Britain. A list of 72 such incinerators was obtained from the Department of the Environment and comprised all incinerators in Great Britain that burn (or burned) household, commercial and/or industrial waste, and for which publicly available information is (or was) available as required by the Control of Pollution Act 1974 and the Environmental Protection Act 1990. It was based on data held by Her Majesty's Inspectorate of Pollution and the waste disposal authorities, and included the full address and postcode of the incinerator, a six-figure grid reference for the site, and information on years of operation and previous use of the site. Checks were made to verify this information using Ordnance Survey maps, the postcode directory and, where possible, by contacting previous operators of the site.

The study was conducted in two stages. In the first a random sample of incinerators was selected, stratified by population size (above/below median population within 3 km). This was done to give sufficient sample size to detect, with 80% power, a relative risk within 3 km of around 1.5 for rare cancers (e.g. larynx). Recent incinerators, i.e. those starting from 1976, were excluded, although one was found later to be within 5 km of an incinerator selected into the sample. Where circles of 7.5 km radius centred on the incinerators overlapped, they were treated as one multisite group, with cases and populations assigned to their nearest incinerator based on the postcode. Three multisite groups (seven incinerators) and nine single incinerators were selected; four incinerators were later added to the three multisite groups as a result of new information received after the sampling had taken place. This included additions to the list of incinerators and revisions of the postcodes. Thus there was a total of 20 incinerators included in the first stage of the study. The remaining 52 incinerators (31 in ten multisite groups and 21 single sites) were included in the second stage. Because a larger population was included in the second stage, statistical power was higher than in the first stage. The location of incinerators is shown in Figure 1.

*Cases, standard rates and expected numbers*

The study is based on postcoded cancer incidence data from the national cancer registration scheme, which, at the time of study, were held for 1974–86 (England), 1974–84 (Wales) and 1975–87 (Scotland). Following a retrospective postcoding exercise undertaken by the Office of Population Censuses and Surveys (OPCS) the level of valid postcoding of cancers in England and Wales 1974–84 ranged from 89.5% (1974) to 98.0% (1984). Postcoding in Scotland, and in England for 1985 and 1986, is in excess of 98%. Table I shows the cancers included in the study and the codes under the 8th and 9th revisions of the International Classification of Disease (ICD) (WHO, 1967, 1978). Bridge-codes were used for cancers in England and Wales to ensure comparability between the two revisions of the ICD (OPCS, 1981). For Scotland, cancers are all coded to the 9th revision.

To calculate national rates cancer incidence for Great Britain was obtained directly from the postcoded SAHSU database. Population data were from the 1981 census small-area statistics. To allow for possible socioeconomic confounding a deprivation score, shown elsewhere to be a powerful predictor of cancer rates across Great Britain (Elliott, 1995), was calculated for each census enumeration district using three variables derived from 1981 census data on unemployment, overcrowding and social class of head of household. Methods used to obtain the deprivation score and for the calculation of expected numbers are given in the Appendix.

For solid tumours and, to a lesser extent, for cancers of the lymphatic and haematopoietic systems a lag period should be assumed between first exposure to a putative cancer-inducing agent and development of clinical disease (Rothman, 1986); lag periods of 10 years for solid cancer cases, and 5 years, are used here. Thus with a 10 year lag only cases occurring at least 10 years after a site began operation are included. In the first stage lag periods for the multisite groups were taken from the most recently started/commissioned incinerator in the whole group. In the second stage data were used more efficiently by taking lag periods from date of start-up of the nearest incinerator. As a check on possible residual confounding, data were also examined for the 'preincinerator' period, i.e. before start-up of a site, which involved differing sets of incinerators than in the lagged analyses because of the different time periods. To avoid contamination of the preincinerator period, data for a site were excluded if it fell within the 7.5 km circle centred on an older (operational) site.

Because of the problems of histology coding for liver cancer, a review of registration was undertaken with the assistance of OPCS and the Information and Statistics Division of the Scottish Health Service. Cancer registries in England and Wales, and in Scotland, were asked to check registration details of all 85 liver cancer cases from 0 to 1 km in the second stage (10 year lag) as well as random samples of 75 cases from 1 to 7.5 km, and a further 75 cases from the rest of Great Britain. Copies of death certificates for these cases were also requested.

**Figure 1** Incinerator sites in Great Britain, first (△) and second (■) stages.

*Statistical methods*

As the study was not done in response to specific claims of cancer excess, there was no prior knowledge of the health statistics around any of the plants. Analyses were performed in two stages to guard against type I error (false positive); significant findings from the first stage were tested again in the second stage, i.e. in a different data set. As noted, the second stage provided greater statistical power as a result of its larger population size and observation period. With inference based on significant ($P<0.05$) findings at both stages, a nominal $P$-value of 0.0025 (i.e. $0.05 \times 0.05$) was used to allow for multiple testing across tumour groups and corresponded to an overall $P$-value of less than 0.05.

For descriptive purposes, observed (O) and expected (E) values, observed/expected ratios (O/E) and their 95% confidence intervals (CIs; calculated assuming a Poisson distribution) are reported for the entire study area (0–7.5 km) and for an area close to the source, arbitrarily chosen at outset to be 0–3 km. Formal tests of significance were based on likelihood ratio tests described by Stone (1988) for decline in risk at some (unspecified) distance from the source. Observed and expected values were obtained for eight bands delimited by circles up to a radius of 7.5 km, chosen at outset to give four near to the source with the remainder enclosing bands of approximately equal area, i.e. radii of 0.5, 1.0, 2.0, 3.0, 4.6, 5.7, 6.7 and 7.5 km. Each likelihood ratio test considers the data from all bands simultaneously to produce a single $P$-value–obtained by comparison with 999 simulated data sets–which in part overcomes problems of inference associated with the arbitrary choice of boundaries. Both unconditional and conditional likelihood tests were performed (Bithell and Stone, 1989; Hills, 1992; Bithell *et al.*, 1994). For the unconditional test the null hypothesis is the same for all sources (incinerators), i.e. constant relative risk of 1.0 (the regional average) in all bands. The data can therefore be aggregated across sources, within bands, and a single likelihood ratio test performed. For the conditional test any differences from the regional averages in overall level of risk up to 7.5 km are corrected with the condition that the sum of the expected values should equal the total number of observed cases within 7.5 km. This gives a null hypothesis that is unique to each source. For this reason the overall (studywide) $P$-value is obtained by carrying out a conditional likelihood ratio test for each source and considering the sum of the maximum likelihood ratios obtained from the individual sources. For cancers with significant ($P<0.05$) findings in both stages, further, post hoc, analysis was carried out to investigate the issue of possible residual confounding.

### Results

Data are presented here for both sexes combined at all ages. Maps, population data and tables giving sex- and age-specific results (<65 and 65+ years) for the single incinerators and incinerator groups are available on request.

*First stage*

At the 1981 census around 890 000 people lived within 0 to 3 km, and 3.3 million within 0 to 7.5 km of the 20 incinerators included in the first stage of the study. In all, 177 252 cancers were recorded from 0 to 7.5 km, of which 69 155 were at ages below 65 years and 108 097 at 65 years and above; 85 113 cases (43 383 males, 41 730 females) were included for the 10 year lag (i.e. 10 or more years since start-up of an incinerator) and 140 010 cases (71 203 males, 68 807 females) for the 5 year lag. Average periods of observation, weighted by population size around each incinerator, were 5.7 and 9.8 years for the 10 year and 5 year lag periods respectively.

Table II shows O/E ratios and 95% CIs for all cancers combined, solid tumours and lymphatic and haematopoietic cancers from 0 to 3 and 0 to 7.5 km. From 0 to 3 km raised O/E ratios and 95% CIs that exclude one (i.e. an excess significant at the 5% level) were found for all cancers combined, stomach, colorectal, liver, lung, bladder, all lymphatic and haematopoietic cancers combined and non-Hodgkin lymphomas; these O/E ratios ranged from 1.05 (lymphatic and haematopoietic) to 1.29 (liver) (Table II).

For the above solid tumours and for all cancers combined results of Stone's unconditional and conditional tests were highly significant. Stone's unconditional test was also significant ($P<0.05$) for non-Hodgkin lymphomas, and it was of borderline significance ($P=0.09$) for all lymphatic and haematopoietic cancers combined (Table II). On the basis of these results, all cancers combined, stomach, colorectal, liver, lung and bladder cancer, as well as non-Hodgkin lymphomas and all lymphatic and haematopoietic cancers combined, were included in the second stage of the study.

Connective tissue cancers were also included in view of a priori interest (Hardell and Sandstrom, 1979; Eriksson *et al.*, 1981; Woods *et al.*, 1987) and the small numbers of these cancers included in the first stage of the study.

*Second stage*

At the 1981 census around 3.4 million people lived from 0 to 3 km and 11.4 million from 0 to 7.5 km from the 52 incinerators included in the second stage of the study. In all, 573 318 cancers were recorded from 0 to 7.5 km, of which

**Table I** Bridge-codes for 8th and 9th revisions of the International Classification of Disease by cancer site

| *Description* | *8th revision* | *9th revision* |
|---|---|---|
| All cancers | 140–207 | 140–208 and 238.6 except 202.2, 202.3, 202.4, 202.5, 202.6 and 202.9 |
| Stomach | 151 | 151 |
| Colorectal | 153 excluding 153.9 and 154 | 153 and 154 |
| Liver | 155 and 197.8 | 155 |
| Nasal and nasopharyngeal | 160 and 147 | 160 and 147 |
| Larynx | 161 | 161 |
| Lung | 162 | 162 |
| Connective | 171 | 171 |
| Bladder | 188 | 188 |
| Lymphatic and haematopoietic | 200–207 | 200–208 and 238.6 except 202.2, 202.3, 202.4, 202.5 202.6 and 202.9 |
| Non-Hodgkin | 200, 202 | 200, 202.0, 202.1, 202.8 |
| Hodgkin | 201 | 201 |
| Multiple myeloma | 203 | 203 and 238.6 |
| Leukaemias | 204–207 | 204–208 |

229 270 were at ages below 65 years and 344 048 at 65 years and above; 354 831 cases (181 118 males, 173 713 females) were included for the 10 year lag and 494 869 cases (253 374 males, 241 495 females) for the 5 year lag. Average periods of observation were 7.0 and 11.0 years for the 10 year and 5 year lag periods respectively.

Table III shows O/E ratios and 95% CIs for all cancers combined, solid tumours, all lymphatic and haematopoietic cancers combined and non-Hodgkin lymphomas from 0 to 3 and 0 to 7.5 km. Raised O/E ratios and 95% CIs that exclude one were found for all cancers, and stomach, colorectal, liver and lung cancers, both from 0 to 3 and 0 to 7.5 km; values of the O/E ratios, ranging (0–3 km) from 1.04 (all cancers and colorectal) to 1.13 (liver), were smaller than in the first stage of the enquiry. For these cancers, results of Stone's unconditional and conditional tests were highly significant, except the conditional test for liver, which was of borderline significance ($P=0.06$).

Data for these cancers for all eight bands are shown in Table IV, including the preincinerator as well as the 10 year lag period. With 10 year lag, the largest O/E ratios were found within 1 km except for colorectal cancer (1–2 km); cumulative O/E ratios at 1 km ranged from 1.04 (colorectal cancer) to 1.37 (liver cancer). Of the population of 342 638 recorded at the 1981 census as resident within 0–1 km of an incinerator site, 46% was in the most deprived quintile of enumeration districts, with only 5.5% in the least deprived. For the 10 year lag period the final column in Table IV gives ratios of expected values adjusted/unadjusted for deprivation. Again, the largest ratios were found from 0 to 1 km and ranged from 1.03 (colorectal cancer) to 1.14 (lung), indicating positive socioeconomic confounding within 1 km for these cancers. Adjustment for deprivation therefore reduced O/E ratios (0–1 km) by from 3% to 14%.

Post hoc examination of data for the preincinerator period gives an estimate of residual confounding in the vicinity of incinerators unrelated to incineration. For stomach and lung cancers, O/E ratios were larger in each band for the preincinerator compared with the 10 year lag period, as they were for all cancers in all but the

**Table II** First stage: observed (O) and expected[a] (E) numbers of incident cases, observed/expected (O/E) ratios, 95% CIs and *P*-values for Stone's unconditional (Un) and conditional (Con) tests for (a) all cancers (including lymphatic and haematopoietic) and solid tumours, 10 year lag (b) lymphatic and haematopoietic cancers, 5 year lag; all ages, both sexes combined

(a)

| | 0–3 km | | | | 0–7.5 km | | | | Stone's P-value[b] | |
|---|---|---|---|---|---|---|---|---|---|---|
| *Cause* | *O* | *E* | *O/E* | *95% CI* | *O* | *E* | *O/E* | *95% CI* | *Un* | *Con* |
| All cancers | 25273 | 23349.08 | 1.08 | 1.07–1.10 | 85113 | 81252.31 | 1.05 | 1.04–1.05 | 0.001 | 0.001 |
| Stomach | 1544 | 1441.42 | 1.07 | 1.02–1.13 | 5081 | 4799.49 | 1.06 | 1.03–1.09 | 0.001 | 0.002 |
| Colorectal | 3175 | 2853.30 | 1.11 | 1.07–1.15 | 10452 | 9907.20 | 1.05 | 1.03–1.08 | 0.001 | 0.001 |
| Liver | 152 | 118.10 | 1.29 | 1.10–1.51 | 448 | 408.50 | 1.10 | 1.00–1.20 | 0.001 | 0.003 |
| Nasal and nasopharyngeal | 50 | 60.56 | 0.83 | 0.63–1.09 | 222 | 210.06 | 1.06 | 0.93–1.21 | 0.828 | |
| Larynx | 240 | 213.87 | 1.12 | 0.99–1.27 | 776 | 720.53 | 1.08 | 1.00–1.16 | 0.201 | |
| Lung | 4982 | 4382.57 | 1.14 | 1.11–1.17 | 16083 | 14637.37 | 1.10 | 1.08–1.12 | 0.001 | 0.001 |
| Connective | 104 | 89.51 | 1.16 | 0.96–1.41 | 334 | 323.29 | 1.03 | 0.93–1.15 | 0.490 | |
| Bladder | 1330 | 1113.54 | 1.19 | 1.13–1.26 | 4242 | 3845.28 | 1.10 | 1.07–1.14 | 0.001 | 0.001 |

(b)

| | 0–3 km | | | | 0–7.5 km | | | | Stone's P-value[b] | |
|---|---|---|---|---|---|---|---|---|---|---|
| *Cause* | *O* | *E* | *O/E* | *95% CI* | *O* | *E* | *O/E* | *95% CI* | *Un* | *Con* |
| Lymphatic and haematopoietic | 2248 | 2144.60 | 1.05 | 1.01–1.09 | 7866 | 7770.99 | 1.01 | 0.99–1.03 | 0.092 | 0.105 |
| All leukaemias | 783 | 803.52 | 0.97 | 0.91–1.05 | 2851 | 2889.30 | 0.99 | 0.95–1.02 | 0.529 | |
| Non-Hodgkin | 782 | 702.26 | 1.11 | 1.04–1.19 | 2689 | 2575.67 | 1.04 | 1.01–1.08 | 0.015 | 0.134 |
| Hodgkin | 244 | 231.59 | 1.05 | 0.93–1.19 | 869 | 843.31 | 1.03 | 0.96–1.10 | 0.360 | |
| Multiple myeloma | 439 | 406.51 | 1.08 | 0.98–1.19 | 1457 | 1459.20 | 1.00 | 0.95–1.05 | 0.269 | |

[a]Expected numbers are adjusted by age, sex, deprivation and region. [b]*P*-values calculated using 999 Monte Carlo simulations.

**Table III** Second stage: observed (O) and expected[a] (E) numbers of cases, observed/expected (O/E) ratios, 95% CIs and *P*-values for Stone's unconditional (Un) and conditional (Con) tests for (a) all cancers (including lymphatic and haematopoietic) and solid tumours, 10 year lag (b) lymphatic and haematopoietic cancers, 5 year lag; all ages, both sexes combined

(a)

| | 0–3 km | | | | 0–7.5 km | | | | Stone's P-value[b] | |
|---|---|---|---|---|---|---|---|---|---|---|
| *Cause* | *O* | *E* | *O/E* | *95% CI* | *O* | *E* | *O/E* | *95% CI* | *Un* | *Con* |
| All cancers | 114394 | 110494.5 | 1.04 | 1.03–1.04 | 354831 | 347500.1 | 1.02 | 1.02–1.02 | 0.001 | 0.001 |
| Stomach | 7496 | 7119.3 | 1.05 | 1.03–1.08 | 22307 | 21685.1 | 1.03 | 1.02–1.04 | 0.001 | 0.001 |
| Colorectal | 13946 | 13423.4 | 1.04 | 1.02–1.06 | 42669 | 42024.7 | 1.02 | 1.01–1.03 | 0.001 | 0.001 |
| Liver | 643 | 569.3 | 1.13 | 1.05–1.22 | 1873 | 1771.1 | 1.06 | 1.01–1.11 | 0.002 | 0.062 |
| Lung | 23309 | 21566.1 | 1.08 | 1.07–1.09 | 70326 | 66421.4 | 1.06 | 1.05–1.07 | 0.001 | 0.001 |
| Connective | 450 | 436.1 | 1.03 | 0.94–1.13 | 1386 | 1388.0 | 1.00 | 0.95–1.05 | 0.452 | |
| Bladder | 4918 | 4864.7 | 1.01 | 0.98–1.04 | 15679 | 15397.8 | 1.02 | 1.00–1.03 | 0.185 | |

(b)

| | 0–3 km | | | | 0–7.5 km | | | | Stone's P-value[b] | |
|---|---|---|---|---|---|---|---|---|---|---|
| *Cause* | *O* | *E* | *O/E* | *95% CI* | *O* | *E* | *O/E* | *95% CI* | *Un* | *Con* |
| Lymphatic and haematopoietic | 7943 | 7845.9 | 1.01 | 0.99–1.03 | 25769 | 25713.7 | 1.00 | 0.99–1.01 | 0.577 | |
| Non-Hodgkin | 2651 | 2563.8 | 1.03 | 1.00–1.07 | 8573 | 8452.6 | 1.01 | 0.99–1.04 | 0.368 | |

[a]Expected numbers are adjusted by age, sex, deprivation and region. [b]*P*-values calculated using 999 Monte Carlo simulations.

innermost band (Table IV), and results of Stone's tests for the preincinerator period were significant ($P<0.05$ to $P=0.001$) (not shown). For colorectal cancer, O/E ratios in the preincinerator period were above one up to 3 km, although the Stone's tests in the preincinerator period were not significant (not shown). For liver cancer, no clear pattern emerged on comparison of the preincinerator and 10 year lag periods (Table IV). Again, results of Stone's tests for the preincinerator period were not significant, although, for liver cancer, these findings were based on a small number of cases (Table IV).

*Further analyses of liver cancer cases (second stage)*

The apparent association with liver cancer was larger than that for the other cancers, and in view of evidence for strong

**Table IV** Second stage: observed (O) numbers of cases, observed/expected (O/E) ratios, cumulative (Cum) O/E ratios and deprivation ratios[a] (DR) by distance for all eight bands for preincinerator and 10 year lag periods for (a) all cancers and (b) stomach cancer, (c) colorectal cancer, (d) liver cancer and (e) lung cancer; all ages, both sexes combined

(a) *All cancers*

| | *Pre* | | | | *10 year lag* | | | |
|---|---|---|---|---|---|---|---|---|
| *Distance (km)* | *O* | *O/E* | *Cum O/E* | *DR* | *O* | *O/E* | *Cum O/E* | *DR* |
| 0.5 | 41 | 0.95 | 0.95 | 1.07 | 2170 | 1.04 | 1.04 | 1.06 |
| 1.0 | 424 | 1.12 | 1.11 | 1.07 | 10137 | 1.07 | 1.06 | 1.05 |
| 2.0 | 2436 | 1.12 | 1.11 | 1.01 | 41855 | 1.04 | 1.04 | 1.04 |
| 3.0 | 3694 | 1.12 | 1.12 | 1.00 | 60232 | 1.03 | 1.04 | 1.03 |
| 4.6 | 5675 | 1.06 | 1.09 | 1.00 | 97893 | 1.01 | 1.02 | 1.02 |
| 5.7 | 3380 | 1.04 | 1.08 | 1.05 | 58153 | 1.02 | 1.02 | 1.01 |
| 6.7 | 4160 | 1.14 | 1.09 | 1.03 | 49008 | 1.01 | 1.02 | 1.01 |
| 7.5 | 3833 | 1.09 | 1.09 | 1.03 | 35383 | 1.01 | 1.02 | 1.00 |

(b)

| | *Pre* | | | | *10 year lag* | | | |
|---|---|---|---|---|---|---|---|---|
| *Distance (km)* | *O* | *O/E* | *Cum O/E* | *DR* | *O* | *O/E* | *Cum O/E* | *DR* |
| 0.5 | 5 | 1.70 | 1.70 | 1.15 | 154 | 1.15 | 1.15 | 1.13 |
| 1.0 | 31 | 1.21 | 1.26 | 1.15 | 704 | 1.13 | 1.14 | 1.11 |
| 2.0 | 169 | 1.18 | 1.19 | 1.03 | 2768 | 1.06 | 1.08 | 1.08 |
| 3.0 | 281 | 1.30 | 1.25 | 1.01 | 3870 | 1.03 | 1.05 | 1.07 |
| 4.6 | 388 | 1.11 | 1.19 | 1.00 | 6107 | 1.00 | 1.03 | 1.04 |
| 5.7 | 240 | 1.09 | 1.16 | 1.08 | 3536 | 1.02 | 1.03 | 1.02 |
| 6.7 | 279 | 1.12 | 1.16 | 1.09 | 3050 | 1.04 | 1.03 | 1.02 |
| 7.5 | 250 | 1.05 | 1.14 | 1.08 | 2118 | 1.02 | 1.03 | 1.00 |

(c)

| | *Pre* | | | | *10 year lag* | | | |
|---|---|---|---|---|---|---|---|---|
| *Distance (km)* | *O* | *O/E* | *Cum O/E* | *DR* | *O* | *O/E* | *Cum O/E* | *DR* |
| 0.5 | 6 | 1.17 | 1.17 | 1.04 | 242 | 0.98 | 0.98 | 1.03 |
| 1.0 | 46 | 1.03 | 1.05 | 1.05 | 1215 | 1.06 | 1.04 | 1.03 |
| 2.0 | 295 | 1.10 | 1.09 | 1.01 | 5209 | 1.07 | 1.06 | 1.02 |
| 3.0 | 435 | 1.05 | 1.07 | 1.00 | 7280 | 1.02 | 1.04 | 1.02 |
| 4.6 | 626 | 0.94 | 1.01 | 1.00 | 11789 | 1.00 | 1.02 | 1.01 |
| 5.7 | 397 | 0.99 | 1.00 | 1.04 | 6928 | 1.01 | 1.02 | 1.00 |
| 6.7 | 513 | 1.15 | 1.03 | 1.03 | 5860 | 1.01 | 1.02 | 1.00 |
| 7.5 | 440 | 1.03 | 1.03 | 1.02 | 4146 | 1.00 | 1.02 | 1.00 |

(d)

| | *Pre* | | | | *10 year lag* | | | |
|---|---|---|---|---|---|---|---|---|
| *Distance (km)* | *O* | *O/E* | *Cum O/E* | *DR* | *O* | *O/E* | *Cum O/E* | *DR* |
| 0.5 | 0 | 0.00 | 0.00 | 1.16 | 13 | 1.12 | 1.12 | 1.16 |
| 1.0 | 0 | 0.00 | 0.00 | 1.17 | 72 | 1.43 | 1.37 | 1.13 |
| 2.0 | 12 | 1.23 | 1.01 | 1.02 | 221 | 1.06 | 1.13 | 1.10 |
| 3.0 | 13 | 0.89 | 0.94 | 1.00 | 337 | 1.13 | 1.13 | 1.08 |
| 4.6 | 34 | 1.46 | 1.18 | 0.99 | 526 | 1.08 | 1.11 | 1.04 |
| 5.7 | 15 | 1.04 | 1.15 | 1.08 | 281 | 0.98 | 1.08 | 1.01 |
| 6.7 | 16 | 0.98 | 1.12 | 1.08 | 242 | 0.98 | 1.06 | 1.02 |
| 7.5 | 16 | 1.02 | 1.10 | 1.06 | 181 | 1.01 | 1.06 | 1.00 |

(e)

| | *Pre* | | | | *10 year lag* | | | |
|---|---|---|---|---|---|---|---|---|
| *Distance (km)* | *O* | *O/E* | *Cum O/E* | *DR* | *O* | *O/E* | *Cum O/E* | *DR* |
| 0.5 | 10 | 1.15 | 1.15 | 1.17 | 479 | 1.13 | 1.13 | 1.16 |
| 1.0 | 92 | 1.18 | 1.18 | 1.18 | 2183 | 1.14 | 1.14 | 1.14 |
| 2.0 | 515 | 1.28 | 1.26 | 1.03 | 8321 | 1.05 | 1.07 | 1.10 |
| 3.0 | 769 | 1.28 | 1.27 | 1.01 | 12326 | 1.09 | 1.08 | 1.08 |
| 4.6 | 1054 | 1.08 | 1.18 | 1.00 | 19051 | 1.03 | 1.06 | 1.04 |
| 5.7 | 692 | 1.14 | 1.17 | 1.09 | 11463 | 1.07 | 1.06 | 1.02 |
| 6.7 | 905 | 1.30 | 1.20 | 1.10 | 9652 | 1.06 | 1.06 | 1.02 |
| 7.5 | 819 | 1.22 | 1.20 | 1.08 | 6851 | 1.04 | 1.06 | 1.00 |

[a]Deprivation ratios are the ratios of expected numbers of cases, adjusted/unadjusted for deprivation.

Table V Second stage, liver cancer: observed (O) numbers of cases, expected values (E) estimated by four different methods, observed–expected ratios (O/E) and cumulative (Cum) O/E ratios, 10year lag; all ages, both sexes combined

| | | $E_0$ (adjusted age, sex, region) | | | E (adjusted age, sex, deprivation, region) | | | $E_1$ (based on incidence of all cancers)[a] | | | $E_2$ (based on incidence of stomach and lung cancer) | | |
|---|---|---|---|---|---|---|---|---|---|---|---|---|---|
| Distance (km) | O | $E_0$ | $O/E_0$ | Cum $O/E_0$ | E | O/E | Cum O/E | $E_1$ | $O/E_1$ | Cum $O/E_1$ | $E_2$ | $O/E_2$ | Cum $O/E_2$ |
| 0.5 | 13 | 10.19 | 1.28 | 1.28 | 11.63 | 1.12 | 1.12 | 11.49 | 1.13 | 1.13 | 12.96 | 1.00 | 1.00 |
| 1.0 | 72 | 44.86 | 1.60 | 0.54 | 50.48 | 1.43 | 1.37 | 53.82 | 1.34 | 1.30 | 57.36 | 1.25 | 1.21 |
| 2.0 | 221 | 190.37 | 1.16 | 1.25 | 208.94 | 1.06 | 1.13 | 221.69 | 1.00 | 1.07 | 220.59 | 1.00 | 1.05 |
| 3.0 | 337 | 277.61 | 1.21 | 1.23 | 298.29 | 1.13 | 1.13 | 317.87 | 1.06 | 1.06 | 329.65 | 1.02 | 1.04 |
| 4.6 | 526 | 468.89 | 1.21 | 1.18 | 486.34 | 1.08 | 1.11 | 516.35 | 1.02 | 1.04 | 510.83 | 1.03 | 1.03 |
| 5.7 | 281 | 283.66 | 0.99 | 1.14 | 288.00 | 0.98 | 1.08 | 306.61 | 0.92 | 1.02 | 301.11 | 0.93 | 1.01 |
| 6.7 | 242 | 242.98 | 1.00 | 1.11 | 247.65 | 0.98 | 1.06 | 259.07 | 0.93 | 1.00 | 254.38 | 0.95 | 1.00 |
| 7.5 | 181 | 179.87 | 1.01 | 1.10 | 179.73 | 1.01 | 1.06 | 186.09 | 0.97 | 1.00 | 182.11 | 0.99 | 1.00 |

[a]Liver cancer excluded.

potential confounding by deprivation in the vicinity of incinerators, and the fact that liver cancer is one of the cancers most strongly related to deprivation (Elliott 1995), possible confounding for liver cancer was examined in further post hoc analyses as shown in Table V. For the eight bands, the table summarises the O/E and cumulative O/E ratios obtained according to four different methods of calculation of expected values. The first set of columns on the left of Table V shows expected values ($E_0$) calculated without adjustment for deprivation. From 0 to 1 km, there was a 54% excess (85 observed, 55.1 expected) and Stone's tests were highly significant (unconditional $P=0.001$, conditional $P=0.007$) (not shown). The second set of columns shows the analysis with adjustment for deprivation; as already noted, from 0 to 1 km, there was a 37% excess (62.1 expected). The third and fourth set of columns show expected values calculated according to the distribution, age and sex stratified, of all cancers combined (liver excluded) and of stomach and lung cancers, which, like liver cancer, are strongly related to deprivation (Elliott, 1995). For liver cancer, comparison with the latter two cancers in particular might be expected to give closer control for confounding by, e.g. sociodemographic or lifestyle factors, than that obtained by use of the deprivation index. Only the conditional version of the Stone's test was carried out for each of the latter two analyses as expected values already sum to the total number of cases within 7.5 km. From 0 to 1 km there was a 30% excess (65.3 expected) and a 21% excess (70.3 expected) in comparison respectively, with all cancers combined and with stomach and lung cancer (Table V). P-values from Stone's test were $P=0.048$ and $P=0.19$ respectively.

The possibility that known risk factors for liver cancer were differentially distributed near incinerators was also examined. Risk factors include hepatitis B infection, which is prevalent in Africa and the Far East, as well as liver cirrhosis related to excess alcohol consumption (Falk, 1982). Commercially available data on alcohol consumption suggested, if anything, a slightly lower prevalence (37.7%) of 'heavy drinking' for the types of areas within 1 km of incinerators, in comparison with Great Britain as a whole (39.4%) (data from CACI). Data on place of birth from the 1981 census for the population living from 0 to 1 km of incinerators showed larger than expected numbers (based on the deprivation profile of the areas) of men and women born in the Indian subcontinent, and men from the Caribbean Commonwealth. Using published data on the mortality of migrants (Marmot *et al.*, 1984; Grulich *et al.*, 1992) this could explain around two or three of the 23 excess liver cancer cases within 0–1 km.

Because routine data overestimate the incidence of primary liver cancer, especially over the age of 65 (Doll and Peto, 1983), the effects of age and the extent of possible misdiagnosis of liver cancer were also examined. Stone's unconditional tests for liver cancer were significant ($P<0.05$) both below 65 years and at age 65 and above. Of 1873 liver cancers registered, with 10 year lag, from 0 to 7.5 km, 82% (1543) were coded to 155.0 (primary liver cancer) compared with 79% for Great Britain. Stone's unconditional test remained significant ($P=0.002$) when restricted to primary liver cancer (155.0) only.

Review of histology coding of liver cancer cases confirmed a substantial level of misdiagnosis and disagreement between registration and death certificate diagnosis, both among cases coded to primary liver cancer (155.0) and for the other ICD codes included for liver cancer (Table I). Of the total of 235 cases reviewed, the diagnoses of three liver cases originally coded to 155.0 were changed by the registries–one was withdrawn as invalid, one was reclassified to cirrhosis and the third to thyroid cancer. The death certificates for two further cases, confirmed as primary liver cancer by the registries concerned, could not be traced. Of the remaining 230 cases, 21 were identified as secondary cancers, either directly by the registry concerned and/or from scrutiny of the death certificates. A further 21 cases were recorded with carcinomatosis (primary unknown) or metastases to liver and 17 others, including five with a death certificate diagnosis of cirrhosis, had no mention of liver cancer on the death certificate. Thus overall, 62/235 (26.8%) of the liver cases either had probable cirrhosis, misdiagnosed primary cancer, confirmed secondary cancers, metastases with unknown primary sites or had death certificates without mention of liver cancer. In addition, for several other cases, it was not possible from the registration details and death certificates to distinguish between primary and secondary liver cancers.

### Discussion

This is the first study systematically to investigate cancer risk related to MSW incineration among the general population. It involved observations on over 14 million people for up to 13 years around 72 MSW incinerators in Great Britain. Previous reports have focused on incinerator workers (Gustavsson, 1989), twinning rates, sex ratios at birth and malformations (Lloyd *et al.*, 1988; Jansson and Voog, 1989; Williams *et al.*, 1992), incinerators of waste solvents and oils (Diggle, 1990; Elliott *et al.*, 1992*b*) or were limited to the study of cancer risk around one or two incinerators only (Diggle, 1990; Hallenbeck *et al.*, 1993; Hoglund and Haglind, 1993). Based on replicated findings in the two stages of the present study, significant results were obtained for all cancers combined, stomach, colorectal, liver and lung cancer. For all other cancers studied, including larynx, nasal and nasopharyngeal cancer, connective tissue (including soft-tissue sarcoma), and non-Hodgkin lymphomas, there was no evidence overall for decline in risk with distance from incinerators.

The significant findings reported here were unlikely to be due to chance, as the design of the study, with replication of findings in two different data sets, guarded against type I error (false positive). However, whereas an observational study of this kind can provide evidence of association, in itself it cannot demonstrate causality–interpretation of the findings is crucially dependent on (well-known) limitations of the data and methods, including possible sources of bias and confounding (Elliott *et al.*, 1992*a,b,c*; Elliott, 1995). A key difficulty is the lack of exposure information. The study includes older incinerators going back to the turn of the century that were likely to have had a very different exposure scenario to the low levels of pollutants found near a modern well-maintained plant (Greim, 1990; Clayton *et al.*, 1991). In the absence of such exposure information, we used a general exposure decline–distance model, equal in all directions, which for any particular incinerator may have been more or less appropriate, depending on stack height, wind patterns, abatement equipment etc.

While errors and biases in small-area studies tend to be conservative, i.e. to lead to negative rather than positive findings (Elliott *et al.*, 1992*b*), confounding can operate powerfully in the opposite direction and lead to positive associations between environmental pollution and disease in the absence of a true (causal) link (Jolley *et al.*, 1992; Elliott, 1995). This is because deprivation tends to be high in polluted areas as well as being strongly predictive of disease occurrence (Jolley *et al.*, 1992; Dolk *et al.*, 1995; Elliott, 1995). Area-based measures of deprivation are predictive of risk factors measured at the individual level such as smoking (Kleinschmidt *et al.*, 1995). Nonetheless, it is possible that some of the association between deprivation and ill health reflects higher pollution levels or some other environmental factor in those areas, so that adjustment for deprivation might result in 'overcontrol' in the analysis (Dolk *et al.*, 1996). In our view, the strong sociodemographic and lifestyle effects associated with deprivation are likely to outweigh any effect of background pollution in those areas. By adjusting for deprivation we are examining for independent associations of disease risk with proximity to the polluting source, over and above any associations with background, sociodemographic and lifestyle factors. In the present study the ratios of expected values adjusted/unadjusted for deprivation indicated positive socioeconomic confounding, especially for all cancers and cancers of the lung, stomach and liver. We, therefore considered the possibility that residual confounding might explain the positive associations between cancer risk and proximity to incinerators.

Thus in the post hoc analyses data for the preincinerator period were examined since, by definition, any excess risk would be unrelated to incineration and could be assumed to reflect residual confounding in the vicinity of incinerators. For lung, stomach and all cancers, the extent of such confounding was sufficient to explain the pattern of risk observed with 10 year lag; comparison of O/E ratios with the ratios of expected values adjusted/unadjusted for deprivation suggested that for those cancers, adjustment controlled only around half of the confounding due to deprivation. For colorectal cancer, the analysis of the preincinerator period was suggestive of residual confounding within 3 km and the estimated relative risk with 10 year lag was lower than for the other cancers, i.e. around 1.04 up to 3 km. For liver cancer O/E ratios adjusted for deprivation were the largest of all cancers examined in either stage of the study, e.g. O/E ratios of 1.91 from 0 to 1 km and 1.29 from 0 to 3 km (first stage) and 1.37 and 1.13 respectively (second stage); the 37% excess from 0 to 1 km in the second stage corresponds to around 0.95 excess cases $10^{-5}$ $year^{-1}$. As noted, liver is one of the cancers most strongly related to deprivation (Elliott, 1995). In view of evidence of important socioeconomic confounding near incinerators possible residual confounding for liver cancer was, therefore, explored further.

In post hoc analyses (second stage) the incidence of liver cancer was compared with that of all cancers combined (excepting liver) and also with stomach and lung cancer in an attempt to achieve closer control for confounding; control for the effects of migration was also achieved to some extent, since both observed and expected numbers were generated by cases who were resident in the area at the time of registration. In these analyses the 37% excess (23 cases) of liver cancer from 0 to 1 km was reduced to 21% (15 cases) against stomach and lung cancer. Analysis by country of birth suggested that no more than two or three excess cases from 0 to 1 km could be explained by the higher than expected number of people living in those areas who were born in the Indian subcontinent or Caribbean Commonwealth – known to have increased risk of liver cancer.

The review of cancer registration details and death certificates confirmed a substantial level of misdiagnosis of primary liver cancer. This is to be investigated further by 'blind' histological review of liver cancer cases in the second stage from 0 to 1 km, as well as random samples of cases from 1 to 7.5 km and from the rest of Great Britain.

In summary, this study of over 14 million people observed for up to 13 years around 72 MSW incinerators in Great Britain found no evidence overall for decline in risk with distance from incinerators for a number of cancers including non-Hodgkin lymphomas and soft-tissue sarcomas. A likely explanation of significant findings for all cancers combined, stomach and lung cancer was residual confounding, which also appeared to explain at least part of the excess risk of liver cancer. For this reason and because of the substantial level of misdiagnosis (mainly secondary tumours) found among registrations and death certificates for liver cancer, further investigation including histological review of the cases is to be done. This should help determine whether or not there is an increase in primary liver cancer in the vicinity of incinerators.

**Acknowledgements**

The Small Area Health Statistics Unit is funded by grants from the Department of Health, Department of the Environment, Health and Safety Executive, Scottish Office Home and Health Department, Welsh Office and Northern Ireland Department of Health and Social Services. We thank the Office of Population, Censuses and Surveys (OPCS) and the Information and Statistics Division of the Scottish Health Service, who made the postcoded cancer data available to us and checked registration details of individual cases; and OPCS and General Register Office (Scotland) for providing copies of death certificates. We are grateful for the efforts of the individual cancer registries that submit data to the national cancer registration scheme, and for their help in checking individual records; and the SAHSU Steering Committee, its scientific members and external advisers, and Professors Peter Smith and Peter Diggle for helpful comments. We should also like to thank other members of the SAHSU team, especially Dr Helen Dolk, Dr Susana Sans and Martine Vrijheid, and Dr Stephanie Coster (Department of the Environment), who provided and checked details of the list of incinerators.

### References

BITHELL JF AND STONE, RA. (1989). On statistical methods for analysing the geographical distribution of cancer cases near nuclear installations. *J. Epidemiol. Community Health*, **43**, 79–85.

BITHELL J, DUTTON S, DRAPER G AND NEARY, N. (1994). Distribution of childhood leukaemias and non-Hodgkin's lymphomas near nuclear installations in England and Wales. *Br. Med. J.*, **309**, 501–505.

BRITISH MEDICAL ASSOCIATION. (1991). *Hazardous Waste and Human Health.* A report from the BMA Professional & Scientific Division. p. 46. Oxford University Press: Oxford.

CLAYTON P, COLEMAN P, LEONARD A, LOADER A, MARLOWE I, MITCHELL D, RICHARDSON S AND SCOTT D. (1991). *Review of Municipal Solid Waste Incineration in the UK.* Report LR 776 (PA). Warren Spring: Stevenage.

DIGGLE PJ. (1990). A point process modelling approach to raised incidence of a rare phenomenon in the vicinity of a prespecified point. *J. R. Stat. Soc. A.*, **153**, 349–362.

DOLK H, MERTENS B, KLEINSCHMIDT I, WALLS P, SHADDICK G AND ELLIOTT P. (1995). A standardisation approach to the control of socio-economic confounding in small area studies of environment and health. *J. Epidemiol. Community Health*, **49**, (suppl. 2), S9–S14.

DOLL R AND PETO R. (1983). Epidemiology of cancer. In *Oxford Textbook of Medicine*, Weatherall DJ, Ledingham JGG, Warrell DA (eds) 4.51-4.78. Oxford University Press: Oxford.

ELLIOTT P. (1995). Small-area studies. In *Environmental Epidemiology: Exposure and Disease*. pp. 187–199. Lewis Publications/ CRC Press: Boca Raton, FL.

ELLIOTT P, WESTLAKE AJ, HILLS M, KLEINSCHMIDT I, RODRIGUES L, McGALE P, MARSHALL K AND ROSE G. (1992*a*). The Small Area Health Statistics Unit: a national facility for investigating health around point sources of environmental pollution in the United Kingdom. *J. Epidemiol. Community Health*, **46**, 345–349.

ELLIOTT P, HILLS M, BERESFORD J, KLEINSCHMIDT I, JOLLEY D, PATTENDEN S, RODRIGUES L, WESTLAKE A AND ROSE G. (1992*b*). Incidence of cancer of the larynx and lung near incinerators of waste solvents and oils in Great Britain. *Lancet*, **339**, 854–858.

ELLIOTT P, KLEINSCHMIDT I AND WESTLAKE A J. (1992*c*). Use of routine data in studies of point sources of environmental pollution. In *Geographical and Environmental Epidemiology: Methods for Small-Area Studies*, Elliott P, Cuzick J, English D, Stern R (eds) pp. 106–114. Oxford University Press: Oxford.

ERIKSSON M, HARDELL L, BERG NO, MOLLER T AND AXELSON O. (1981). Soft-tissue sarcomas and exposure to chemical substances: a case-reference study. *Br. J. Ind. Med.*, **38**, 27–33.

FALK H. (1982). Liver. In *Cancer Epidemiology and Prevention*, Schottenfeld D, Fraumeni J (eds) pp. 668–682. W B Saunders: Philadelphia.

FINGERHUT MA, HALPERIN WE, MARLOW DA, PIACITELLI LA, HONCHAR PA, SWEENEY MH, GREIFE AL, DILL PA, STEENLAND K AND SURUDA AJ. (1991). Cancer mortality in workers exposed to 2,3,7,8-tetrachlorodibenzo-p-dioxin. *N. Engl. J. Med.*, **342**, 212–218.

GOUGH M. (1991). Human health effects: what the data indicate. *Sci. Total Environ.*, **104**, 129–158.

GREIM H. (1990). Toxicological evaluation of emissions from modern municipal waste incinerators. *Chemosphere*, **20**, 317–331.

GRULICH A E, SWERDLOW AJ, HEAD J AND MARMOT MG. (1992). Cancer mortality in African and Caribbean migrants to England and Wales. *Br. J. Cancer*, **66**, 905–911.

GUSTAVSSON P. (1989). Mortality among workers at a municipal waste incinerator. *Am. J. Ind. Med.*, **15**, 245–253.

HALLENBECK WH, BREEN S P AND BRENNIMAN G R. (1993). Cancer risk assessment for the inhalation of metals from municipal solid waste incinerators impacting Chicago. *Bull. Environ. Contam. Toxicol.*, **51**, 165–170.

HARDELL L AND SANDSTROM A. (1979). Case–control study: soft tissue sarcomas and exposure to phenoxyacetic acids or chlorophenols. *Br. J. Cancer*, **39**, 711–717.

HATTEMER-FREY HA AND TRAVIS CC. (1991). *Health Effects of Municipal Waste Incineration*. CRC Press: Boca Raton, FL.

HILLS M. (1992). Some comments on methods for investigating disease risk around a point source. In *Geographical and Environmental Epidemiology: Methods for Small-Area Studies*, Elliott P, Cuzick J, English D, Stern R (eds) pp. 231–237. Oxford University Press: Oxford.

HOGLUND D AND HAGLIND P. (1993). Health effects of air pollution from waste incineration. *Proceedings of Fifth International Conference of International Society for Environmental Epidemiology*, Stockholm, Aug 15–18. p. 80 (Abstract P1:2).

INTERNATIONAL AGENCY FOR RESEARCH ON CANCER WORKING GROUP. (1982). *Chemicals, Industrial Processes and Industries Associated with Cancer in Humans*. IARC Monographs on the evaluation of carcinogenic risk of chemicals to man, vols 1 to 29, suppl. 4. IARC: Lyon.

INTERNATIONAL AGENCY FOR RESEARCH ON CANCER. (1984). *Polynuclear Aromatic Compounds*. IARC Monographs on the evaluation of carcinogenic risk of chemicals to man, vol. 34. IARC: Lyon.

INTERNATIONAL AGENCY FOR RESEARCH ON CANCER. (1987). *Overall Evaluations of Carcinogenicity: an Updating of IARC Monographs Vols 1 to 42*. IARC Monographs on the evaluation of carcinogenic risk to humans, suppl. 7. IARC: Lyon.

JANSSON B AND VOOG L. (1989). Dioxin from Swedish municipal incinerators and the occurrence of cleft lip and palate malformations. *Int. J. Environ. Studies*, **34**, 99–104.

JOLLEY D, JARMAN B AND ELLIOTT P. (1992). Socio-economic confounding. In *Geographical and Environmental Epidemiology: Methods for Small-Area Studies*, Elliott P, Cuzick J, English D, Stern R (eds) pp. 115–124. Oxford University Press: Oxford.

KLEINSCHMIDT I, HILLS M AND ELLIOTT P. (1995). Smoking behaviour can be predicted by neighbourhood deprivation measures. *J. Epidemiol. Community Health*, **49**, (suppl. 2) S72–S77.

KOCIBA RJ, KEYES DG, BEYER JE, CARREON RM, WADE CE, DITTENBER DA, KALNINS RP, FRAUSON LE, PARK CN, BARNARD SD, HUMMEL RA AND HUMISTON CG. (1978). Results of a two-year chronic toxicity and oncogenicity study of 2,3,7,8-tetrachlorodibenzo-p-dioxin in rats. *Oncol. Appl. Pharmacol.*, **46**, 279–303.

LLOYD OM, LLOYD MM, WILLIAMS FLR AND LAWSON A. (1988). Twinning in human populations and cattle exposed to air pollution from incinerators. *Br. J. Ind. Med.*, **45**, 556–560.

MANZ A, BERGER J, DWYER JH, FLESCH-JANYS D, NAGEL S AND WALTSGOTT H. (1991). Cancer mortality among workers in chemical plant contaminated with dioxin. *Lancet*, **338**, 959–964.

MARMOT MG, ADELSTEIN AM AND BULUSU L. (1984). *Immigrant Mortality in England and Wales 1970–78. Causes of Death by Country of Birth*. Studies on Medical and Population Subjects, No. 47. pp. 28–45. HMSO: London.

OFFICE OF POPULATION CENSUSES AND SURVEYS. (1981). *Mortality Statistics. Comparison of 8th and 9th revisions of the International Classification of Diseases (ICD)*. Series DH1 No. 10. HMSO: London.

ROTHMAN KJ. (1986). *Modern Epidemiology*. p. 58. Little, Brown: Boston.

ROYAL COMMISSION ON ENVIRONMENTAL POLLUTION. (1993). *Incineration of Waste*. Seventeenth Report (Chairman: Houghton J). HMSO: London.

SKENE SA, DEWHURST IC AND GREENBERG M. (1989). Polychlorinated dibenzo-p-dioxins and polychlorinated dibenzofurans: the risks to human health. A review. *Hum. Toxicol.*, **8**, 173–203.

STONE RA. (1988). Investigations of excess environmental risks around putative sources: statistical problems and a proposed test. *Stat. Med.*, **7**, 649–660.

TRAVIS CC AND HATTEMER-FREY H A. (1989). Human exposure to dioxin from municipal solid waste incinerators. *Waste Management*, **9**, 151–156.

WILLIAMS FLR, LAWSON AB AND LLOYD OL. (1992). Low sex ratios of births in areas at risk from incinerators, as shown by geographical analysis and 3-dimensional mapping. *Int. J. Epidemiol.*, **21**, 311–319.

WOODS JS, POLISSAR L, SEVERSON RK, HEUSER LS AND KULANDER BG. (1987). Soft tissue sarcoma and non-Hodgkin's lymphoma in relation to phenoxyherbicide and chlorinated phenol exposure in Western Washington. *J. Natl Cancer. Inst.*, **78**, 899–910.

WORLD HEALTH ORGANIZATION. (1967). *Manual of the International Statistical Classification of Diseases, Injuries and Causes of Death* (8th revision conference). WHO: Geneva.

WORLD HEALTH ORGANIZATION. (1978). *Manual of the International Statistical Classification of Diseases, Injuries and Causes of Death* (9th revision conference). WHO: Geneva.

WORLD HEALTH ORGANIZATION. (1988). *Emissions of Heavy Metal and PaH Compounds from Municipal Solid Waste Incinerators: Control Technology and Health Effects*. Report on a WHO meeting, Florence 12–16 October 1987. WHO: Copenhagen.

ZOBER A, MESSERER P AND HUBER P. (1990). Thirty-four year mortality follow-up of BASF employees exposed to 2,3,7,8-TCDD after the 1953 incident. *Int. Arch. Occup. Environ. Health*, **62**, 139–157.

**Appendix**

*Deprivation score, and calculation of expected values*

Each of the three variables making up the deprivation score (unemployment, overcrowding, social class of head of household) was standardised across Great Britain to have zero mean and unit variance. A $z$-score for each variable was obtained for each enumeration district and its deprivation score was calculated as the sum of the three $z$-scores. Deprivation scores were then grouped into national quintiles. A small (sixth) stratum included those enumeration districts where data were insufficient to provide a score.

National rates ($r_{ijkl}$) were calculated for each calendar year for 216 strata defined by deprivation score (six groups), sex and age (18 × 5 year groups).

| | | |
|---|---|---|
| $r_{ijkl}$ | $i=1, \ldots, 6$ | deprivation |
| | $j=1,2$ | sex |
| | $k=1, \ldots, 18$ | age group |
| | $l=1, \ldots, 14$ | calendar year (1974–87) |

When data for a particular country were unavailable (e.g. Wales 1985–87), data for the nearest available year (e.g. 1984) were used in the estimation of national rates.

Expected numbers for the study areas were then obtained. First, numbers standardised for age and deprivation were calculated separately for males and females and each calendar year.

$$E_{jl} = \sum_{ik} E_{ijkl} = \sum_{ik} \left[ r_{ijkl} \times p_{ijkl} \right]$$

where $p_{ijkl}$ is the population of the study area, stratified by deprivation, sex, age and calendar year. Adjustment was then made for regional differences in incidence rate or levels of completeness of registration and postcoding, by multiplying these expected values by sex- and year-specific standardised incidence ratios for the region, adjusted for deprivation.

$$E'_{jl} = E_{jl} \times \frac{O^r_{jl}}{E^r_{jl}}$$

where $O^r{}_{ij}$ and $E^r{}_{jl}$ are the observed and expected numbers for the entire region, stratified by sex and calendar year.

Finally, these numbers were summed up over calendar years for males, females and for both sexes combined. For example, for both sexes combined: